# Stable and High-Throughput Single-Cell Sorting of Food Bacteria Using Spatiotemporal Video-Enhanced Raman Tweezers

**DOI:** 10.3390/foods15122208

**Published:** 2026-06-18

**Authors:** Yi Sun, Zhipeng Li, Hua Xia, Kaier Yang, Feng Gao, Yingxiao Peng, Xiangyun Ma, Qifeng Li

**Affiliations:** 1 State Key Laboratory of Precision Measurement Technology and Instruments, Tianjin University, Tianjin 300072, China; yisun33@tju.edu.cn (Y.S.); 18323092063@163.com (Z.L.); xiahua@tju.edu.cn (H.X.); 3123005504@tju.edu.cn (K.Y.); gaofeng9777@tju.edu.cn (F.G.); 1023202085@tju.edu.cn (Y.P.); 2 School of Precision Instrument and Opto-Electronics Engineering, Tianjin University, Tianjin 300072, China

**Keywords:** foodborne pathogens, detection and analysis methods, Raman tweezers spectroscopy, microfluidics, food safety

## Abstract

Rapid detection of foodborne pathogenic and spoilage microorganisms is critical for ensuring food safety and quality in liquid matrices. While Raman tweezers spectroscopy (RTS) enables label-free single-cell analysis, its application in high-throughput inline inspection faces a fundamental bottleneck: high flow rates required for efficiency induce severe motion blur and low signal-to-noise ratios (SNR), which blind automated control systems and destabilize optical trapping. To overcome this, we present a Spatiotemporal Video-Enhanced Raman Tweezers (SVERT) system integrating a deceleration-optimized microfluidic chip with a deep learning-based visual feedback loop. We propose a Local–Global Unified Denoising Network (LGU-Net) tailored to recover high-fidelity bacterial structures from low-SNR video streams, achieving a deterministic processing latency of ~0.49 ms. Experimental results demonstrate that SVERT improves the optical trapping success rate from 21.27% ± 2% to 91.47% ± 1.8% compared to raw video input, enabling a four-fold increase in spectral acquisition efficiency. Leveraging the acquired high-quality dataset, we achieved a classification accuracy of 96.74% across four bacterial species of relevance to food safety and quality. Crucially, we validated the system’s practical robustness by successfully isolating and tracking trace *E. coli* in an unpurified commercial beverage. This capability to effectively mitigate natural background interference demonstrates the system’s promising potential to be expanded for broader applications in liquid food safety screening.

## 1. Introduction

Food spoilage and contamination caused by microorganisms pose a severe challenge to global food safety, quality, and shelf life, resulting in massive economic losses [1,2]. In nutrient-rich liquid food matrices, microbial contamination primarily manifests as either visible food spoilage (organoleptic deterioration) or hidden pathogenic threats. Crucially, foodborne pathogens such as *Staphylococcus aureus* can proliferate and cause severe infections or intoxications without altering the food’s organoleptic properties. Rapid and early detection of these vegetative cells before significant proliferation occurs is crucial for preventing subsequent contamination in the food supply chain [3,4]. While conventional culture-based techniques remain the gold standard, they are inherently time-consuming and resource-intensive, often preventing timely intervention [5,6,7].

To address these limitations, Raman tweezers spectroscopy (RTS) has emerged as a powerful alternative for label-free, real-time single-cell analysis [8,9]. By integrating RTS with microfluidic platforms, it becomes possible to precisely manipulate and analyze individual bacteria within a controlled fluid environment, offering a theoretical foundation for inline, high-throughput screening of microbial contaminants in liquid food streams [10,11,12].

**Figure 1:**
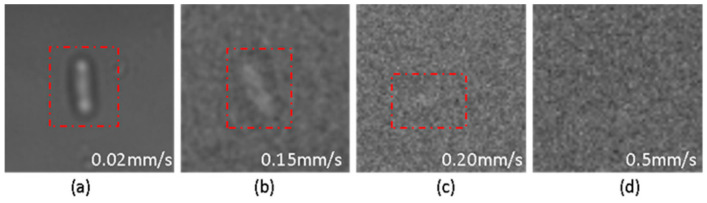
The impact of increased flow rates on bacterial imaging: from (**a**–**d**), as the sample input speed increases, the exposure time decreases, and the bacterial structure is progressively obscured by noise, eventually leading to an inability to accurately resolve the bacterial positions in the field of view. The red dashed boxes indicate the location of the target bacterium.

However, realizing high-throughput RTS in practice faces a fundamental trade-off between flow speed and optical trapping stability [13,14]. To accelerate detection, high-flow rates are often employed, yet the necessitated short exposure times drastically reduce the signal-to-noise ratio (SNR) and degrade image clarity. This visual impairment severely compromises the ability to stably capture and track bacterial positions, ultimately hindering effective localization in real-time Raman-based detection systems [15,16,17]. As illustrated in Figure 1, increasing the sample flow rate leads to progressively shorter exposure times and more severe noise interference, causing bacterial structures to become increasingly obscured and eventually indistinguishable in the field of view. Crucially, this visual degradation blinds the automated localization algorithms required to guide the optical tweezers. When the system cannot precisely locate the fast-moving bacterium within the millisecond-scale engagement window, the optical trap fails to engage, resulting in a dramatic drop in capture efficiency and analysis throughput.

Addressing this issue requires image enhancement strategies that are both structure-preserving and computationally efficient [18]. Recent advancements in video denoising have focused on improving noise removal in dynamic sequences while preserving motion details. One key approach integrates spatial and temporal information, using three-dimensional convolutions to effectively handle dynamic scenes. Another development involves deformable convolutional kernels that address pixel misalignment in moving sequences. Self-supervised learning techniques and multi-scale architectures have also emerged, improving denoising efficiency and feature preservation [19,20]. Recent studies have also demonstrated the power of deep learning in enhancing the accuracy of bacterial identification via Raman spectroscopy [21]. Despite these advancements, existing methods fail to reconcile precision with speed: they either over-smooth faint biological details or incur computational latencies that exceed the optical trap’s millisecond-scale feedback loop. This gap necessitates a real-time spatiotemporal solution capable of functionally restoring the vision of optical tweezers without compromising high-throughput performance.

In this work, we developed a spatiotemporal video-enhanced Raman tweezers (SVERT) system that bridges this gap by integrating a custom-designed Local–Global Unified Denoising Network (LGU-Net) into the optical trapping control loop. The model utilizes a dual-parallel architecture, comprising three modules: Global Feature Modeling (GFM), Local Feature Enhancement (LFE), and Unified Feature Integration (UFI). It is optimized using a hybrid loss function designed to improve noise suppression, bacterial edge preservation, and texture recovery. Unlike general-purpose models, LGU-Net is tailored for microfluidic imaging, achieving a deterministic processing latency of 0.49–2.68 ms, which allows for real-time synchronization with high-speed image acquisition. This paper validates the proposed system in a bacterial detection scenario relevant to food safety. Furthermore, while previous optical trapping systems are predominantly validated in ideal laboratory buffers, we explicitly evaluated our SVERT system in a real-world liquid food matrix to simulate trace-level microbial contamination using *Escherichia coli*, a model foodborne pathogen. Comprehensive comparisons against traditional filters and modern video denoising baselines demonstrate that our spatiotemporal architecture can handle natural matrix interference, offering insights for its future adaptation in food safety and quality monitoring applications.

## 2. Materials and Methods

### 2.1. Culture Conditions

In this study, four distinct bacterial species were selected for evaluation: *Bacillus cereus* (*B. cereus*), *Bacillus subtilis* (*B. subtilis*), *Escherichia coli* (*E. coli*), and *Staphylococcus aureus* (*S. aureus*). These bacterial strains were obtained from the China General Microbiological Culture Collection Center (CGMCC) and were cultured in Tryptic Soy broth at 37 °C for 12 h under aerobic conditions. After incubation, the cultures were centrifuged at 5000× *g* for 10 min to pellet the bacteria, and the supernatants were discarded. The bacterial pellets were then washed twice with sterile phosphate-buffered saline (PBS) to remove any remaining culture medium and resuspended in PBS for Raman spectral analysis. The PBS used for dilution was preheated in a water bath. The prepared bacterial samples were subjected to gradient dilution using PBS to obtain the required concentration for microfluidic injection.

To further assess the practical applicability and high-sensitivity performance of the SVERT system in authentic food environments, an artificial contamination assay was conducted using commercial coconut water as a representative complex liquid matrix. Specifically, a precise volume of the serially diluted *E. coli* PBS suspension prepared above was spiked into commercial coconut water (obtained from a local supermarket) to yield a trace concentration of approximately 50 CFU/mL [22,23]. To emulate a trace-level pathogenic contamination scenario with low target abundance, the artificially contaminated matrix was briefly incubated at 37 °C for 4 h. Following this short-term incubation, the complex, unpurified mixture was injected directly into the microfluidic chip. By deliberately bypassing conventional pre-enrichment and isolation steps, this rigorous setup explicitly validates the robustness of the LGU-Net in sustaining stable, real-time optical trapping against the strong optical interference and low signal-to-noise ratios typical of complex food matrices.

### 2.2. Microfluidic System

The microfluidic device was fabricated from PDMS (10:1 mixing ratio, Sylgard 184) and designed specifically to achieve ideal flow deceleration for high-throughput detection. Finite element simulation (Figure S1, Supporting Information) confirms that the chip geometry creates a deceleration zone, thereby achieving a 2 to 5 times longer imaging window at flow rates exceeding 0.1 mm/s compared to a standard straight channel. Based on these simulations, the channel design was optimized with a buffer radius of 0.15 mm and 9 pairs of microstructures. The detailed geometric parameters of the microfluidic chip design are summarized in Table S1 (Supporting Information). Prior to the experiment, the microfluidic chip was cleaned with 70% (*v*/*v*) ethanol solution and pre-filled with PBS buffer to prevent air bubble formation. The bacterial suspension was stably injected into the chip using an automated syringe pump at a constant flow rate of 0.18 mm/s.

### 2.3. Raman Spectral Acquisition

The experimental setup consisted of a custom-built confocal Raman spectroscopy system integrated with optical tweezers, all mounted on a motorized microscope stage. For optical trapping and spectroscopy, a 633 nm diode laser was employed for both bacterial trapping and signal excitation. The laser power was stabilized at about 10 mW, focused onto the sample with an approximate spot size of 1 μm. Bacterial cells were directly observed through a 60 × oil-immersion objective lens with a NA of 0.9, and the resulting Raman signals were detected by a cooled CCD. Raman spectra were recorded in the 400–2000 cm^−1^ fingerprint region with a spectral resolution of 2 cm^−1^, with an integration time of 1 s per spectrum, and the entire collection process was automated via a custom LabVIEW program (JCOPTIX, China). Concurrently, Video-Enhanced Imaging was achieved using a high-frame-rate camera to capture the rapid movement dynamics of the bacteria. The system acquired 16-bit gray video images at a resolution of 512 × 128, with the frame rate fixed at a high speed of 140 frames per second (FPS).

### 2.4. Paired Video Dataset Construction

As no public dataset exists for bacterial video denoising under microfluidic flow, we constructed a dedicated paired dataset from experimentally acquired data. Video sequences of these bacteria were captured within the microfluidic chip and processed into image sequences. In total, we constructed a dataset comprising 800 paired groups (200 groups for each bacterial species). Each group consists of a 5-frame sequence of high-quality, 16-bit grayscale images (resolution: 96 × 72) paired with a corresponding noisy sequence. To ensure rigorous evaluation, the complete dataset was randomly partitioned into training, validation, and test sets with a ratio of 8:1:1. The process involved two key stages to ensure the noise characteristics were physically accurate:

1. Ground Truth (GT) Acquisition: We captured 800 clean, 5-frame sequences at 16-bit grayscale and 96 × 72 resolution of four bacterial strains: *E. coli*, *S. aureus*, *B. subtilis*, and *B. cereus*. These were captured under low flow conditions of less than 0.05 mm/s. These conditions ensured high-SNR images, which served as our reference clean data.

2. Realistic Noise Modeling: To replicate the noise in high-flow scenarios, we physically extracted a real noise profile from our imaging system. This was done by recording background videos (without bacteria) using short exposure times ranging from 6.67 to 13.33 ms to replicate the signal degradation observed at high flow rates exceeding 0.5 mm/s. Temporal noise was isolated from the static background using frame-to-frame differencing. The final noisy training sequences were generated by scaling this real noise with coefficients (representing σ = 0.05 to 0.55) and adding it to the GT sequences. Obtaining perfectly aligned, clean and noisy pairs of fast-moving bacteria under actual high-flow conditions is physically unfeasible. This background extraction approach is valid because the noise characteristics are dominated by the imaging hardware and short exposure flow conditions. Extracting the real system noise profile ensures the physical authenticity of the training data.

### 2.5. LGU-Net Architecture

**Figure 2:**
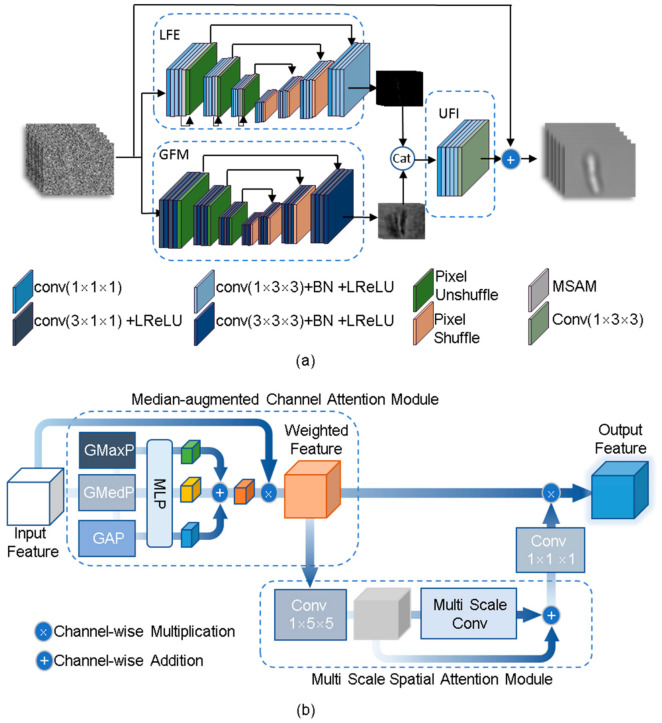
The architecture of the proposed LGU-Net. (**a**) The overall framework of the dual-branch network. In the figure, conv(t × h × w) represents a convolution kernel, Batch Normalization (BN) indicates batch normalization, and LReLU stands for the Leaky ReLU activation function. The meanings of the rectangular blocks are defined at the bottom. (**b**) The detailed structure of the Median-augmented Spatial Attention Module (MSAM), illustrating the median-augmented channel attention and multi-scale spatial attention sub-modules.

To overcome the limitations of existing denoisers in recovering clean, structurally faithful images from low-SNR bacterial videos, we developed LGU-Net. The overall architecture, depicted in Figure 2a, is predicated on the necessity to jointly model fine-grained spatial details and long-range spatiotemporal context to effectively denoise weak, fast-moving targets. LGU-Net comprises three core components: the LFE branch, the GFM branch, and the UFI module.

The LFE branch is designed to extract high-quality spatial details from individual frames, addressing the challenges of motion blur and weak texture signals in high-speed imaging. It adopts a 3D convolution-based encoder–decoder architecture that focuses specifically on intra-frame features, utilizing 3D convolution kernels such as 1 × 1 × 1 and 1 × 3 × 3. A key element within this branch is the integration of a Median-augmented Spatial Attention Module (MSAM), detailed in Figure 2b. While existing standard attention mechanisms like CBAM conventionally rely solely on Global Average Pooling (GAP) and Global Max (GMaxP) pooling, such approaches are easily distorted by the extreme outlier noise prevalent in high-flow microfluidics.

To specifically address this, the channel attention sub-module of MSAM introduces a structural innovation by incorporating Global Median Pooling (GMedP) alongside traditional pooling methods. This inclusion offers robust statistical modeling that mitigates extreme noise outliers, allowing the network to preserve biological structures more effectively than standard average pooling methods. Furthermore, compared to existing sequential video denoising networks that stack deep 3D convolutions and incur high processing delays, the dual-parallel layout of the GFM and LFE branches is a tailored architectural novelty. This parallel design functionally decouples spatial and temporal processing, strictly guaranteeing the deterministic ultra-low latency (~0.49 ms) indispensable for the optical trapping feedback loop. Concurrently, the spatial attention sub-module employs multi-scale depthwise convolutions to capture a wide range of spatial dependencies, from fine edges to broader textures. Finally, all downsampling and upsampling operations within the LFE branch are handled by the network to prevent information loss associated with standard pooling.

The GFM module is responsible for capturing long-range temporal dependencies and learning spatiotemporal representations from input video sequences. It adopts a 3D convolution-based encoder–decoder architecture that jointly models spatial and temporal features in a unified framework. The encoder begins with a 3D convolutional layer that expands the input channel dimension, followed by three consecutive spatiotemporal convolutional blocks. Each block consists of 3 × 1 × 1 and 3 × 3 × 3 convolutional kernels, along with batch normalization and Leaky Rectified Linear Unit (LReLU) activation, enabling the network to slide across both spatial and temporal axes and extract motion-aware features. To avoid information loss during resolution reduction, the GFM module replaces conventional pooling or strided convolutions with a Pixel Unshuffle operation, which rearranges spatial positions into channel depth, thereby preserving local structures. The decoder mirrors the encoder design and uses Pixel Shuffle to recover the original spatial resolution by rearranging channels back into spatial positions. This encoding–decoding strategy allows the GFM module to effectively capture frame-to-frame continuity while minimizing structural distortion, leading to robust denoising performance under dynamic and noisy imaging conditions.

The LFE and GFM branches produce complementary features, with the LFE focusing on high-resolution spatial details and the GFM on spatiotemporal context. The UFI module is designed to consolidate these two distinct representations. As shown in the overall architecture, the outputs from both branches are first concatenated and then processed through a simple convolutional block to effectively align and fuse the features. Finally, this fused feature map is added to the original input image via a residual connection. This last step ensures that the final output retains fine structural details from the input, preventing over-smoothing and leading to the final, clean image sequence.

The LGU-Net model was implemented using the PyTorch 1.12.1 deep learning framework and trained on an NVIDIA Tesla A100 GPU. This computational power was essential to match the high-throughput data acquisition rates of our microfluidic system. We employed the Adam optimizer with an initial learning rate of 0.0016 and used Xavier initialization for the network weights. The model was trained for 600 epochs in total, with the learning rate scheduled to be halved every 40 epochs. During training, input samples consisted of 5 consecutive frames, with a batch size of 18. All input pixel values were normalized prior to training.

To comprehensively guide the model towards high-quality output, a hybrid loss function was employed. The hybrid loss ($L_{\mathrm{Hybrid}}$) is a weighted sum of four components: Mean Squared Error ($L_{\mathrm{MSE}}$), Structural Similarity ($L_{\mathrm{SSIM}}$), Perceptual Loss ($L_{\mathrm{Perc}}$), and Total Variation Loss ($L_{\mathrm{TV}}$). The overall function is defined as Equation (1):

(1) $$L_{\mathrm{Hybrid}}=\lambda_{\mathrm{MSE}}L_{\mathrm{MSE}}+\lambda_{\mathrm{SSIM}}L_{\mathrm{SSIM}}+\lambda_{\mathrm{Perc}}L_{\mathrm{Perc}}+\lambda_{\mathrm{TV}}L_{\mathrm{TV}}$$

The weights were empirically set to balance these objectives. $L_{\mathrm{MSE}}$ is the primary driver for pixel-level fidelity, weighted at $\lambda_{\mathrm{MSE}}$ = 0.88 to reduce overall noise. $L_{\mathrm{SSIM}}$ is included with $\lambda_{\mathrm{SSIM}}$ = 0.1 to maintain structural integrity and contrast. To improve perceptual visual quality, $L_{\mathrm{Perc}}$ ($\lambda_{\mathrm{Perc}}$ = 0.01) is used to align high-level features. Finally, *L_TV_* ($\lambda_{\mathrm{TV}}$ = 0.01) acts as a regularization term to smooth the image and reduce artifacts while retaining essential details.

To quantitatively evaluate the performance of the proposed denoising model, two standard image quality metrics were employed: Peak Signal-to-Noise Ratio (PSNR) and the Structural Similarity Index (SSIM). PSNR measures the pixel-level fidelity between the denoised image and the ground-truth, while SSIM assesses the perceptual similarity in terms of structure, luminance, and contrast.

To ensure a strictly fair and fully controlled comparison, the Efficient Multi-Stage Video Denoising (EMVD) network [24] was trained from scratch on the exact same custom paired dataset and under analogous optimization conditions, including identical data normalization, learning rate scheduling, and epoch settings, as our LGU-Net.

### 2.6. Spectral Classification Methodology

To prepare the data for classification, the acquired Raman spectra from 400 to 2000 cm^−1^ were first preprocessed. Each spectrum was first processed by vector normalization to eliminate intensity variations. No baseline correction or smoothing was applied, allowing the network to learn directly from the normalized raw spectral features. A 1D Convolutional Neural Network (CNN) was then designed and employed for the four-class classification task, inspired by established architectures for spectral analysis. The model’s architecture, trained to identify *B. cereus*, *B. subtilis*, *E. coli*, and *S. aureus*, was constructed as follows: The input was passed through a 1D convolutional layer with 16 filters and a kernel size of 3, followed by a BN layer and a ReLU activation function. A second convolutional block with 32 filters and a kernel size of 3, along with BN and ReLU, was then applied. After this, a Global Average Pooling (GAP) layer was used to compress the features into a fixed-length vector. This vector was passed through two consecutive dense (FC) layers with 128 and 64 neurons, respectively, each followed by a ReLU activation. The final FC layer consisted of 4 neurons, corresponding to the four bacterial classes, and used a SoftMax activation function to output the final classification probabilities. The model was trained using the Adam optimizer with an initial learning rate of 0.001 and a batch size of 32 for 100 epochs.

The spectral dataset consists of 400 spectra, comprising 50 successful capturing events per species divided into two independent experimental batches. This dataset was randomly partitioned into a training set (80%) and a test set (20%). Due to the meticulous nature of real-time single-cell optical manipulation, the data acquisition was objectively conducted across multiple independent measurement days and separate experimental sessions. This multi-session strategy ensures that day-to-day and batch-to-batch operational variances are intrinsically captured within the dataset, preventing model overestimation. The classification performance, as evaluated on the independent test set, is presented in the confusion matrix in Section 3. Nevertheless, it is important to note that a random train and test split was applied after pooling the spectral data. More advanced cross-validation protocols, such as leave-one-day-out, leave-one-batch-out, or validation on a completely independent external dataset, were not performed in this work. Consequently, the current classification results primarily reflect the repeatability of our prototype under controlled laboratory conditions rather than its cross-platform generalization capability. The quantitative biological metrics reported in this study, including the optical trapping success rate and Raman classification accuracy, represent the average values derived from two independent experimental batches. These results are expressed as mean ± standard deviation (SD) to reflect measurement repeatability. Deep learning evaluation metrics are reported as the average values computed over the entire independent test set.

## 3. Results

To validate the effectiveness of the proposed LGU-Net model, we first conducted a quantitative comparison against three traditional baseline denoising methods: Mean filtering, Bilateral filtering, and Non-Local Means (NLM) filtering, as well as a deep learning-based video denoising network, EMVD. The evaluation was performed on our test set across six different noise intensities, ranging from σ = 0.05 to 0.55. The quantitative results, measured by PSNR and SSIM, are summarized in Table S2. It is evident that our LGU-Net consistently achieves the highest scores across all noise levels. For instance, at a moderate noise level of σ = 0.25, LGU-Net surpasses the second-best performer, EMVD, by a margin of 1.12 dB in PSNR and 0.0283 in SSIM. Notably, when compared to the EMVD network, LGU-Net exhibits superior robustness, particularly under severe noise conditions. As the noise intensity increases to σ = 0.45, EMVD suffers a significant performance drop (PSNR: 27.14 dB, SSIM: 0.8906), whereas LGU-Net maintains a high restoration quality (PSNR: 30.49 dB, SSIM: 0.9347). This widening performance gap at extreme noise intensities underscores that our custom-designed spatiotemporal architecture is effective at isolating weak biological signals from complex dynamic noise, outperforming general-purpose video denoising networks.

**Figure 3:**
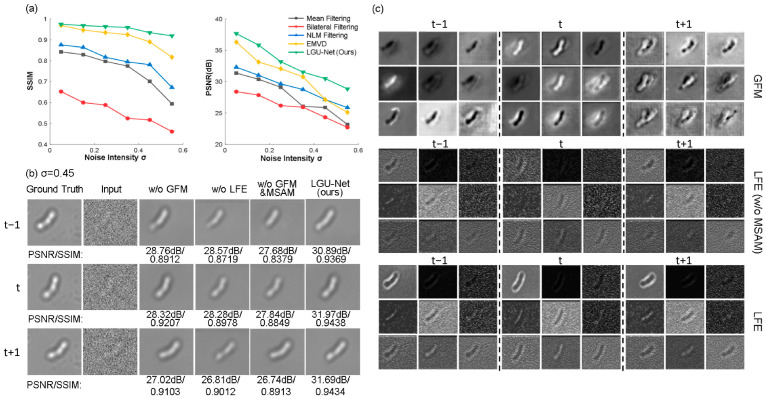
Comprehensive evaluation of LGU-Net performance. (**a**) Quantitative comparison against traditional denoising methods (Mean, Bilateral, and NLM filtering) and the deep learning-based EMVD network across varying noise intensities (σ = 0.05–0.55). The line plots of SSIM and PSNR demonstrate that LGU-Net (green line) consistently outperforms all other evaluated traditional techniques. (**b**) Qualitative ablation study results at a high noise intensity (σ = 0.45). The visual comparison displays the Ground Truth, Noisy Input, and denoising outputs from model variants lacking specific modules (GFM, LFE, MSAM), contrasting with the full LGU-Net model. The PSNR/SSIM scores are shown below the image. (**c**) Visualization of intermediate feature maps from the GFM and LFE branches.

The superiority of our method is systematically evaluated in Figure 3. As shown in Figure 3a, the quantitative trends of PSNR and SSIM clearly demonstrate that LGU-Net (green line) maintains a substantial performance advantage over all traditional methods and the EMVD network across the entire range of noise intensities. This quantitative lead is visually corroborated by the qualitative results presented in Figure 3b. Under a representative high-noise condition (σ = 0.45), the output from the model without the LFE branch appears blurry and lacks textural definition, while the model lacking the GFM branch struggles with noise suppression. In sharp contrast, the full LGU-Net effectively reconstructs the bacteria, preserving the sharpest edges and the most complete structural integrity. This confirms that our deep learning-based approach, with its unified local–global feature modeling, is far more effective at separating the bacterial signal from complex noise than traditional filter-based techniques and general-purpose learning models.

We performed a series of ablation studies to isolate the contribution of individual modules (Table S3, Supporting Information). Removing the LFE branch caused the most significant deterioration in PSNR (3.08 dB), highlighting its dominance in capturing high-fidelity spatial features. Similarly, the exclusion of the GFM branch resulted in a notable 2.31 dB drop, confirming the necessity of temporal context modeling. Crucially, removing the MSAM further attenuated performance, proving its vital role in amplifying the branch’s sensitivity to local structural details.

To provide interpretability for the network’s operation, Figure 3c visualizes the intermediate feature maps. It is evident that the GFM integrates temporal information from adjacent frames (t − 1, t + 1) to model structural continuity. Conversely, the LFE specializes in extracting spatial boundaries and local textures from the current frame (t). By comparison, maps from the LFE without MSAM appear sparse and diffuse, visually validating that MSAM significantly enhances the extraction of fine-grained local features.

Beyond restoration fidelity, computational efficiency is a critical determinant for the feasibility of closed-loop optical trapping. To verify the real-time capability, we evaluated the inference latency of LGU-Net on an NVIDIA Tesla A100 GPU. As summarized in Table 1, the average inference time per frame is 0.49 ms for a 96 × 72 input resolution and 2.68 ms for a 512 × 128 resolution. Considering the high-speed camera operates at 140 FPS (with a ~7.1 ms frame interval), the total processing time remains significantly below the integration window. This ultra-low latency ensures that denoised coordinates are fed back to the optical tweezers within a single frame cycle, effectively eliminating target loss caused by computational lag and enabling stable manipulation under high-throughput conditions.

**Figure 4:**
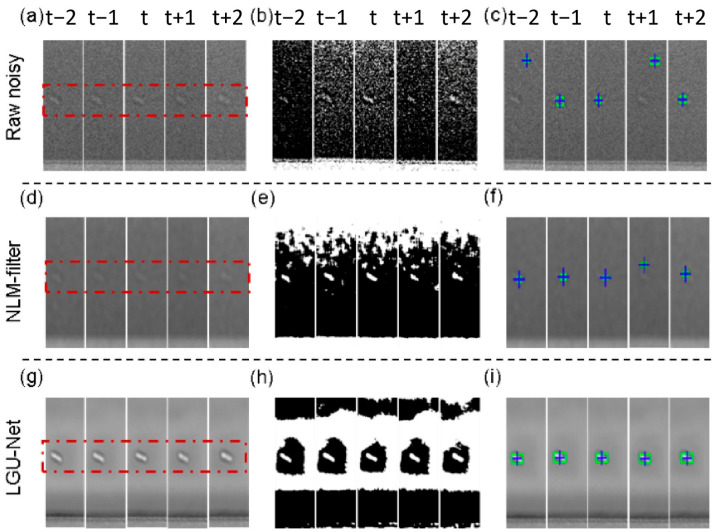
Impact of denoising on downstream bacterial localization in real-world experiments. The figure compares the localization pipeline performance across three processing methods: Raw noisy input; NLM-filtered input; and LGU-Net-denoised input. (**a**,**d**,**g**) Five consecutive frames from the input video sequence processed by each method. (**b**,**e**,**h**) The corresponding intermediate binary masks generated by threshold segmentation. Note that only the LGU-Net output (**h**) yields clear, continuous shapes. (**c**,**f**,**i**) The final localization results, where the detected contour is marked by a green border and the centroid by a blue cross. LGU-Net (**i**) achieves the most stable and accurate tracking. The red dashed boxes in (**a**,**d**,**g**) highlight the region of interest containing the target bacterium across the consecutive frames.

To validate the efficacy of LGU-Net in experimental settings, we assessed its performance on a critical downstream task: bacterial localization, which is a strict prerequisite for stable optical trapping. Figure 4 compares the localization precision across raw, NLM-filtered, and LGU-Net-denoised inputs, demonstrating the model’s practical robustness in complex microfluidic environments.

As shown in the noisy raw sequence in Figure 4a, the low SNR causes the subsequent threshold segmentation (b) to fail in generating a coherent binary map. This results in unstable and largely erroneous localizations, as seen in Figure 4c. Although the classical NLM filter (d) offers a degree of visual improvement, it struggles to fully suppress background fluctuations, leading to noisy and fragmented segmentation masks (e) and inconsistent detection results (f). In contrast, LGU-Net (g) demonstrates superior robustness. It effectively suppresses noise to produce a clean background while preserving the complete bacterial morphology and sharp contours. This high-quality restoration allows the same thresholding step to generate near-ideal binary masks (h), which in turn enables the localization algorithm to stably and accurately pinpoint the bacterium’s centroid and contour across the entire sequence (i). Unlike NLM, which processes frames independently and leads to flickering artifacts, LGU-Net maintains temporal consistency, ensuring the centroid trajectory remains smooth and trackable.

To demonstrate the practical deployment potential of the SVERT system beyond ideal laboratory buffers, we evaluated its video enhancement capabilities in a real-world complex food matrix. *E. coli* cells were spiked into commercial coconut water and incubated to simulate trace-level pathogenic contamination—a scenario relevant to food safety screening where pathogens may be present at low concentrations without visible signs of food deterioration. Unlike clear PBS buffers, coconut water contains abundant biomacromolecules that induce strong optical scattering and complex background interference, severely exacerbating the visual degradation under high-throughput flow conditions.

**Figure 5:**
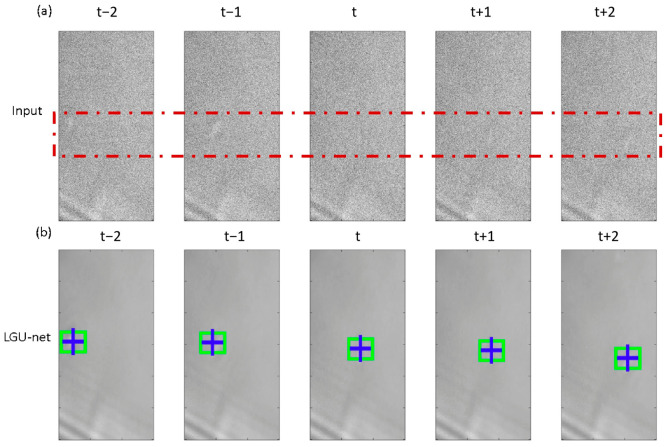
Visual restoration of a single *E. coli* bacterium within a complex food matrix (coconut water) under high-flow conditions. (**a**) Raw input image sequence across consecutive frames (t − 2 to t + 2). (**b**) The corresponding denoised sequence processed by LGU-Net without any task-specific retraining. The detected contour is marked by a green border and the centroid by a blue cross, demonstrating stable and accurate tracking.

As depicted in the raw video frames (Figure 5a), the target bacterium is completely camouflaged by the dense, fluctuating background noise and the dynamic refractive index variations inherent to the coconut water matrix, rendering standard optical tracking impossible. After processing with LGU-Net (Figure 5b), the complex background interference and flow-induced artifacts are effectively suppressed. The network successfully resolves the bacterium, restoring its morphological integrity with high contrast across consecutive frames (t − 2 to t + 2). This demonstrates the generalization capability of our model, indicating that LGU-Net can translate from controlled buffer environments to optically challenging liquid food matrices. By reliably mitigating the severe optical scattering inherent to a real food matrix, LGU-Net ensures the continuous visibility of trace bacterial contaminants, thereby enabling robust downstream optical trapping and providing a critical foundation for real-world food safety screening.

## 4. Discussion

The high-accuracy bacterial identification using Raman spectroscopy heavily relies on the robust localization achieved by LGU-Net. The impact of our denoising method on the system’s practical performance is quantitatively summarized in Table S4 (Supporting Information). The localization loss rate, which indicates the percentage of frames where the algorithm failed to identify the bacterium, plummeted from 78.73% ± 2.0% for raw video to only 8.53% ± 1.8% when using LGU-Net. This dramatic stabilization directly translated into a substantial gain in system throughput: in a standardized evaluation trial, successful Raman spectral acquisitions from optically trapped bacteria surged from merely 11 to 46 out of 50 attempts (yielding the 91.47% ± 1.8% average trapping success rate).

**Figure 6:**
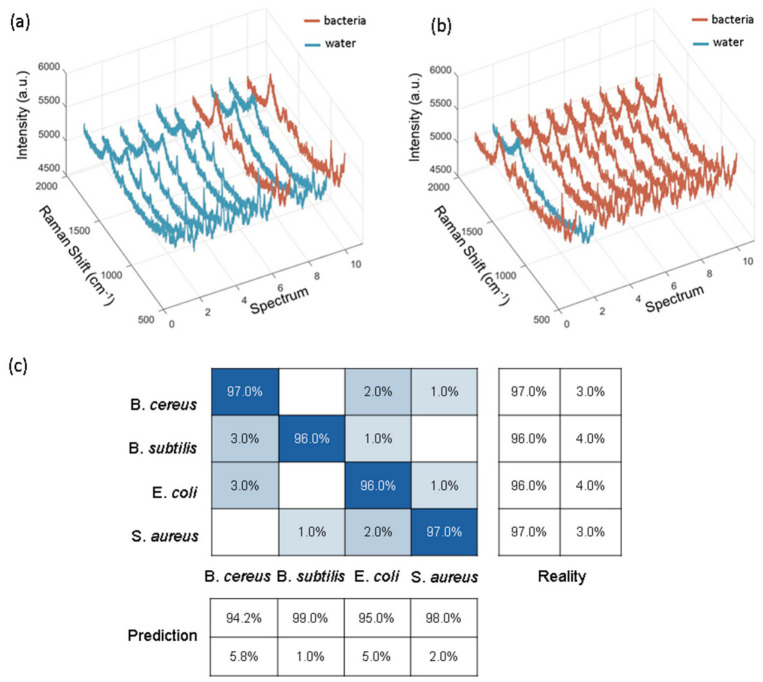
Performance comparison of bacterial trapping success demonstrated by spectral acquisition. (**a**) Spectra acquired using raw video guidance. Due to unstable trapping, the system primarily captured background water signals (blue); only two successful bacterial Raman spectra (brown) were obtained out of ten attempts, indicating a low success rate. (**b**) Spectra acquired using the LGU-Net guided system. Under identical conditions, the system successfully captured nine high-quality bacterial Raman spectra (brown) out of ten attempts, demonstrating a significantly improved success rate and signal stability. (**c**) Confusion matrix of the four-class bacterial spectral classification model.

To visualize this critical improvement in acquisition efficiency, Figure 6a,b presents 3D plots of the spectral data collected from 10 consecutive trapping attempts. In Figure 6a, which corresponds to the system operating on raw video, the optical trap struggled to hold the target, resulting in the capture of mostly background water signals (blue); only two bacterial spectra (brown) were successfully acquired out of ten attempts. In sharp contrast, Figure 6b demonstrates the performance of the system guided by LGU-Net. Under identical experimental conditions, nine high-quality bacterial Raman spectra (brown) were successfully acquired out of ten attempts. These well-resolved spectral profiles and the high success rate visually corroborate the quantitative findings in Table S4 (Supporting Information), demonstrating that LGU-Net is not merely a perceptual enhancement tool, but a functionally critical component that ensures the robustness and practical utility of the entire real-time single-bacterial detection system.

A 1D Convolutional Neural Network was subsequently employed for the four-class classification of the acquired bacterial Raman spectra. The definitive performance of the classifier, evaluated on the test set, is presented in the confusion matrix in Figure 6c. The model demonstrated classification capability, achieving an overall accuracy of 96.74%. This is reflected in the high number of correct predictions, with per-class accuracies of 96.00% or 97.00% for all four species. The performance is further quantified by excellent per-class metrics. Recall rates were high for all species, while precision values were equally strong, reaching up to 99.0% for *B. subtilis*. The few misclassifications primarily occurred between the morphologically similar *Bacillus* species. This pattern underscores the sensitivity of our Raman spectral model, as it is discerning enough to capture the subtle molecular differences between these closely related species, rather than confounding them with more distantly related ones like *E. coli* or *S. aureus*. Overall, these results confirm that our complete video-enhanced Raman tweezers system can achieve rapid and highly accurate identification of single bacteria.

To contextualize the practical utility of the SVERT framework in food safety applications, a comprehensive comparison against alternative single-cell technologies [25,26,27] is provided in Table S5 (Supporting Information). High-throughput fluorescence platforms are inherently constrained by their reliance on target labeling and multistep matrix clearing. Conversely, conventional Raman tweezers eliminate labels but suffer from severe throughput bottlenecks in dynamic flows due to noise-induced tracking failures. SVERT effectively bridges this gap. By leveraging the real-time spatiotemporal denoising of LGU-Net, the system computationally bypasses severe matrix scattering, maintaining stable trapping directly within unpurified beverages. Consequently, SVERT preserves the non-invasive, high-viability advantages of Raman spectroscopy while upgrading throughput to a level viable for practical screening.

Crucially, the macroscopic detection capability in continuous-flow optical trapping is a dynamic threshold governed by a fundamental throughput-sensitivity trade-off. While lower flow velocities extend the observation window to capture rarer cells, higher rates maximize screening throughput at the expense of capture probability. To strike a practical balance, our system was operated at a stable flow velocity of 0.18 mm/s. Under this condition, the volumetric flow rate—bolstered by the 91.47% ± 1.8% trapping success rate restored by LGU-Net—mathematically supports an estimated operational detection level of approximately 50 CFU/mL. While this fluid dynamic validation confirms that SVERT successfully bridges single-cell physical resolution and macroscopic trace detection without relying on tedious 24–48 h pre-enrichment, this 50 CFU/mL threshold serves strictly as an operational estimate under current microfluidic conditions, rather than a formally determined analytical limit of detection. Furthermore, because the SVERT platform operates conceptually as a discrete single-cell trapping and counting system rather than a macroscopic bulk chemical assay, its Limit of Quantification (LOQ) is operationally synchronized with this detection level. Any target concentration lower than this threshold falls below the statistical interception probability required for reliable continuous-flow volumetric sampling.

Finally, while tracking trace bacteria in unpurified coconut water rigorously validates the system’s robustness against non-ideal matrix scattering, analyzing highly diverse mixed microbial communities represents the next critical frontier. Natural microbial contamination in food matrices involves dynamically interacting species, encompassing both spoilage organisms and potential pathogens. Building upon the spatiotemporal denoising foundation established here, integrating multi-target tracking algorithms and multiplexed Raman spectral unmixing to simultaneously classify interacting strains remains a primary objective for our future studies.

## 5. Conclusions

We have developed and validated a SVERT system, successfully overcoming a critical bottleneck in real-time microfluidic analysis: the severe video noise and motion blur that otherwise compromise reliable optical trapping and subsequent identification. By integrating a novel spatiotemporal denoising network, LGU-Net, the system provides high-fidelity visual guidance for precise optical manipulation under low-SNR conditions. Our results demonstrate that LGU-Net effectively preserves fine-grained structural features of bacteria while suppressing complex background noise, consistently outperforming traditional filtering methods and modern deep-learning baselines. With a deterministic inference latency of 0.49–2.68 ms, the network ensures seamless real-time synchronization with high-speed imaging.

The implementation of the SVERT framework significantly enhanced the system’s robustness, increasing the optical trapping success rate from 21.27% ± 2.0% in raw video to 91.47% ± 1.8%. This stable trapping enabled the acquisition of high-quality Raman spectra for four bacterial species of relevance to food safety and quality—including spoilage-associated bacilli and foodborne pathogenic bacteria—achieving a classification accuracy of 96.74%. Crucially, the practical deployment potential of the system was further proven in a real-world liquid beverage matrix. By effectively mitigating natural background fluctuations without any pre-enrichment, LGU-Net enabled stable optical tracking of trace bacteria outside of ideal laboratory buffers. These findings confirm that video enhancement is a vital prerequisite for extending Raman tweezers from basic biophysics to high-throughput inline food safety and quality monitoring applications. Future work will focus on further optimizing the network for multi-target tracking and expanding its application to a wider variety of complex food matrices, ultimately contributing to more robust early-warning strategies for food safety.

## Figures and Tables

**Table 1:** Computational complexity and inference latency of LGU-Net.

Input Resolution	Parameters (M)	GFLOPs	Inference Latency (ms)
96 × 72	2.87 M	8.14	0.49
512 × 128	2.87 M	77.21	2.68

## Data Availability

The original contributions presented in this study are included in the article/Supplementary Materials. Further inquiries can be directed to the corresponding authors.

## Supplementary Materials

The following supporting information can be downloaded at https://www.mdpi.com/article/10.3390/foods15122208/s1, Figure S1: Design and simulation of the microfluidic chip; Table S1: Geometric parameters of the PDMS microfluidic chip; Table S2: Quantitative comparison of denoising results between LGU-Net and other image denoising methods; Table S3: Ablation study on the key components of LGU-Net (at σ = 0.45); Table S4: Quantitative Impact of LGU-Net on Localization and Spectral Acquisition; Table S5: Detailed comparison of SVERT with alternative single-cell technologies for food spoilage detection.
