# Supplementary material for: Stable and High-Throughput Single-Cell Sorting of Food Bacteria Using Spatiotemporal Video-Enhanced Raman Tweezers
Source: Foods. 2026 Jun 18;15(12):2208. doi: 10.3390/foods15122208 (PMC13298320; doi:10.3390/foods15122208)
Supplement: Supplementary file 1 [file foods-15-02208-s001.zip › foods-4309218-supplementary.pdf]

Supporting Information

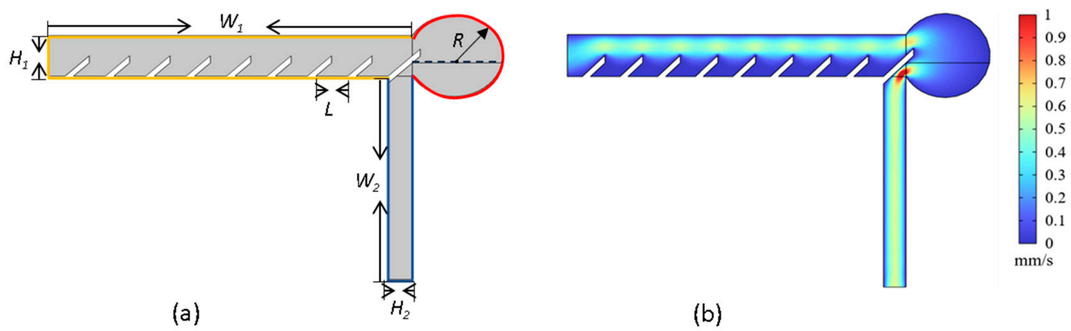

Supplementary Figure S1. Design and simulation of the microfluidic chip. (a) Schematic diagram of the chip structure, illustrating the key geometric parameters (W_1, H_1, R, etc.) and the microstructure array designed for flow deceleration. (b) Finite element simulation results showing the flow velocity distribution. The color gradient (red to blue) indicates the velocity magnitude (mm/s), clearly highlighting the deceleration zone created by the widening channel and microstructures, which extends the effective imaging window for bacterial trapping.

Supplementary Table S1. Geometric parameters of the PDMS microfluidic chip.

**Parameter**	**Value**	**Unit**	**Description**
W_1	0.7	mm	Fluid inlet channel length
H_1	100	μm	Fluid inlet channel height
D_1	50	μm	Microfluidic chip depth
R	0.15	mm	Buffer zone radius
H_2	50	μm	Fluid outlet channel width
W_2	0.5	mm	Fluid outlet channel length
N	9	—	Number of microstructures
L	$\frac{2W_1}{3(N-1)}$	mm	Microstructure spacing

Supplementary Table S2. Quantitative comparison of denoising results between LGU-Net and other image denoising methods.

Noise Intensity (σ)	Method	Mean Filtering	Bilateral Filtering	NLM Filtering	EMVD	LGU-Net
0.05	PSNR	31.37	28.41	32.31	36.32	37.68
	SSIM	0.8428	0.6528	0.8763	0.9692	0.9749
0.15	PSNR	30.39	27.86	31.02	33.14	35.84
	SSIM	0.8293	0.6001	0.8640	0.9471	0.9687
0.25	PSNR	29.11	26.19	29.64	32.07	33.19
	SSIM	0.7971	0.5884	0.8173	0.9352	0.9635
0.35	PSNR	26.06	25.93	28.75	30.78	31.53
	SSIM	0.7749	0.5246	0.7947	0.9255	0.9597
0.45	PSNR	25.89	24.32	27.14	27.14	30.49
	SSIM	0.7017	0.5171	0.7815	0.8906	0.9347
0.55	PSNR	23.18	22.72	25.87	25.12	28.84
	SSIM	0.5942	0.4614	0.6724	0.8173	0.9196

Supplementary Table S3. Ablation study on the key components of LGU-Net (at σ=0.45).

Model Configuration	PSNR (dB)	SSIM
Full LGU-Net (Ours)	30.49	0.9347
w/o LFE module	27.41	0.8912
w/o GFM module	28.18	0.9007
w/o GFM & MSAM	26.89	0.8873

Supplementary Table S4. Quantitative Impact of LGU-Net on Localization and Spectral Acquisition

Evaluation Metric	Raw Video	NLM Filter	LGU-Net (Ours)
Localization Loss Rate (%)	78.73	62.95	8.53
Spectral Acquisition Success (Count / 50)	11	—	46

Table S5. Detailed comparison of SVERT with alternative single-cell technologies for food spoilage detection

Technology	Labeling	Sensitivity	Throughput	Cell Viability	Sample-Preparation Workflow
Flow Cytometry/ FACS	Requires (Fluorescent/Ab)	High (> 10^3 CFU/mL)	10^3–10^4 cells/s	Shear stress risk	Complex
Droplet Microfluidics	Requires (Fluorogenic)	High (Single-cell after incubation)	10^2–10^3 droplets/s	Recovery reduces viability	Moderate
Conventional Raman Tweezers	Label-free	Single-cell (In pure buffers)	<22% capture success in flow	non-destructive	Minimal
SVERT System (This Work)	Label-free	High single-cell level (~50 CFU/mL in raw matrices)	91.47% ± 1.8% capture success in flow	low-power laser fast feedback	Minimal
